# In silico modeling of the interaction between TEX19 and LIRE1, and analysis of *TEX19* gene missense SNPs

**DOI:** 10.1002/mgg3.1707

**Published:** 2021-05-26

**Authors:** Faisal A. Alzahrani, Yousef MohammedRabaa Hawsawi, Hisham N. Altayeb, Naif O. Alsiwiehri, Othman R. Alzahrani, Hanan E. Alatwi, Osama M. Al‐Amer, Suliman Alomar, Lamjed Mansour

**Affiliations:** ^1^ Department of Biochemistry Faculty of Science Embryonic Stem Cell Unit King Fahad Center for Medical Research King Abdulaziz University Jeddah Saudi Arabia; ^2^ Research Center at King Faisal Specialist Hospital and Research Center Jeddah Saudi Arabia; ^3^ College of Medicine Al‐Faisal University Riyadh Saudi Arabia; ^4^ Department of Clinical Laboratory Science Faculty of Applied Medical Science Taif University Taif Saudi Arabia; ^5^ Department of Biology Faculty of Sciences University of Tabuk Tabuk Saudi Arabia; ^6^ Genome and Biotechnology Unit Faculty of Science University of Tabuk Tabuk Saudi Arabia; ^7^ Department of Medical Laboratory Technology Faculty of Applied Medical Sciences University of Tabouk Tabuk Saudi Arabia; ^8^ Doping Research Chair Department of Zoology College of Science King Saud University Riyadh Saudi Arabia; ^9^ Department of Zoology College of Science King Saud University Riyadh Saudi Arabia

**Keywords:** LINE‐1, MD simulation, molecular docking, SNPs analysis, TEX19

## Abstract

**Background:**

Testis expressed 19 (*TEX19*) is a specific human stem cell gene identified as cancer‐testis antigen (CTA), which emerged as a potential therapeutic drug target. TEX19.1, a mouse paralog of human TEX19, can interact with LINE‐1 retrotransposable element ORF1 protein (LIRE1) and subsequently restrict mobilization of LINE‐1 elements in the genome.

**Aim:**

This study aimed to predict the interaction of TEX19 with LIRE1 and analyze *TEX19* missense polymorphisms. TEX19 model was generated using I‐TASSER and the interaction between TEX19 and LIRE1 was studied using the HADDOCK software.

**Methods:**

The stability of the docking formed complex was studied through the molecular dynamic simulation using GROMACS. Missense SNPs (n=102) of *TEX19* were screened for their potential effects on protein structure and function using different software.

**Results:**

Outcomes of this study revealed amino acids that potentially stabilize the predicted interaction interface between TEX19 and LIRE1. Of these SNPs, 37 were predicted to play a probably damaging role for the protein, three of them (F35S, P61R, and E55L) located at the binding site of LIRE1 and could disturb this binding affinity.

**Conclusion:**

This information can be verified by further in vitro and in vivo experimentations and could be exploited for potential therapeutic targets.

## Graphical abstract

interaction pattern between TEX19 and LIRE1 proteins Formation of Van der Waals, electrostatic interactions and hydrogen bonding and formation of TEX19 and LIRE1complex

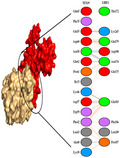

## 1 INTRODUCTION

Cancer/testis (CT) genes are mainly expressed in the testis with a significant upregulation during oncogenesis (Wang et al., 2016) Testis‐expressed 19 (*TEX19*) (OMIM# 615647) is one such mammalian‐specific CT genes that is unique for humans and expressed in adult testis and undifferentiated embryonic stem cells and primordial germ cells (Kuntz et al., 2008; Wang et al., 2001). This gene was duplicated in mouse and rat genomes giving rise to TEX19.1 and TEX19.2 paralogs. Among these paralogs, mouse *Tex19.1* is more similar to human *TEX19* and both genes are expressed throughout pluripotent cycle and their expression is lost when pluripotent stem cells differentiate (Hawsawi et al., 2018; Kuntz et al., 2008). Multiple sequence alignment of TEX19 proteins resulted in two conserved domains, which do not share homologies with known proteins. Therefore, it was unable to predict their functions (Kuntz et al., 2008). In two separate studies, Feichtinger et al. (2012) and Planells‐Palop et al., 2017, studied the expression profiles of human meiotic genes in different types of cancer and conducted meta‐analyses of clinical data sets, however, the role of human TEX19 in cancer was not well reflected both studies (Feichtinger et al., 2012; Planells‐Palop et al., 2017).

Mice with TEX19 double knockout (TEX19DKO) or single TEX19.1KO exhibited a fully penetrant phenotype with impaired spermatogenesis, testis degeneration, small testes (Yang et al., 2010), in oogenesis (Reichmann et al., 2020) defects in meiotic chromosome synapsis, persistence of DNA double‐strand breaks during meiosis, lack of post‐meiotic germ cells, and upregulation of MMERVK10C expression (Ollinger et al., 2008; Tarabay et al., 2013, 2017). However, TEX19.2KO mice presented only a subtle phenotype with discrete seminiferous tubule degeneration in adult male testes (Hawsawi et al., 2020; Tarabay et al., 2017). TEX19.1 is the only transcripts present in developing and adult ovaries as well as in the placenta and TEX19.1KO mouse embryos exhibit intrauterine growth retardation and have small placentas due to reduced number of spongiotrophoblast, glycogen trophoblast and sinusoidal trophoblast giant cells (Reichmann et al., 2013; Tarabay et al., 2013). TEX19 was also identified for its role in the progression of bladder and ovarian cancer and was considered as a potential immunotherapeutic target for cancer treatment (Xu et al., 2020; Zhong et al., 2016).

Retrotransposons are mobile genetic elements which act as significant driver of the evolution of the mammalian genome, but their mobilization can also make the genome vulnerable to genetic disorders and cancers (Al‐Amer et al., 2020; Alsohime et al., 2021; Garcia‐Perez et al., 2016; Kotb et al., 2018). In humans, the majorities of retrotransposition events are activated by long interspersed element class 1 (known as LINE‐1 or L1) (OMIM# 151626) gene which encodes ORF1 protein (also known as LIRE1 which stands for LINE‐1 retrotransposable element ORF1 protein) (Beck et al., 2011). The human TEX19 protein has been shown experimentally to interact with human LINE‐1 ORF1p and promotes polyubiquitylation of hL1‐ORF1p as it restricts mobilization of both human LINE‐1 (MacLennan et al., 2017).

LIRE1 is a nucleic acid‐binding protein that plays an essential role in the retrotransposition of LINE‐1 elements in the genome (Martin & Bushman, 2001). To maintain stability of mammalian genomes and minimizing incidence of mutation and cancer, our cells release factors to restrict the mobilization of L1 through binding and subsequently inhibiting LIRE1. Among these factors, *TEX19*.*1* can act as retrotransposon inhibitor gene which suppresses L1 expression in mice spermatocytes (Reichmann et al., 2012). Mice TEX19.1 protein can interact with LIRE1, thereby restricting mobilization of LINE‐1 retrotransposons in the developing germline (MacLennan et al., 2017). Unlike mice, little is known regarding the interaction between TEX19 with LIRE1 and the effect of missense polymorphisms on this interaction. The main protein interacting partner of TEX19.1 in vivo is Ubr2 (MacLennan et al., 2017; Reichmann et al., 2020; Yang et al., 2010) and the human TEX19 also interacts with UBR2 (Reichmann et al., 2020), and Ubr2 also physically interacts with LINE‐1 ORF1p (MacLennan et al., 2017). Accordingly, there is potentially a trimeric complex between TEX19.1, Ubr2 and LINE‐1 ORF1p.

Previously, we reported several SNPs that associated with cancer (Alzahrani et al., 2020; Hawsawi et al., 2019; Semlali, Almutairi, et al., 2018; Semlali, Parine, et al., 2018). This *in* *silico* study aimed to predict the interaction of TEX19 with LIRE1 and the role of *TEX19* gene polymorphisms in the stability of produced protein and the interaction with LIRE1.

## 2 MATERIALS AND METHODS

### 2.1 Protein structures and homology modeling

The protein sequence of the TEX19 was downloaded from NCBI (ID: NP_997342.1). The 3D structure of TEX19 protein was predicted by using the Iterative‐Threading ASSEmbly Refinement (I‐TASSER) server (Yang & Zhang, 2015). This method generated five models, and the best one was selected based on the C‐Score, which is a measure to observe the quality of resulting models showed the correlation quality of the model prediction results. C‐score is typically in the range of (−5, 2). A C‐score of higher value signifies a model with high confidence and vice versa. The model selected with C‐score (−5.0) was further subjected to molecular dynamic simulation to remove any steric clashes and get a stable structure (Yang et al., 2015). To validate the TEX19 model, a PROSA statistic was used. PROSA is a web‐based interactive software application which shows the energy plots and scores. It aids in identifying the potential problems spotted model structure of the protein. It has a full application in evaluating errors in 3D models of protein. The crystal structure of LINE‐1 protein (PDB ID: 2W7A) was obtained RCSB PDB database (Berman et al., 2002).

### 2.2 Docking

Protein–protein interaction study was performed by High Ambiguity Driven protein‐protein DOCKing (HADDOCK) software (Dominguez et al., 2003). Proteins were uploaded for docking at HDDOCK server and all parameters were kept as default.

### 2.3 Molecular dynamic (MD) simulation

MD simulation of the interested TEX19 and LIRE1 complex was carried out using GROMACS package (Hess et al., 2008), CHARMM 36 force‐field (Huang et al., 2017), and the TIP3P water model (Price & Brooks, 2004). The system charges were then neutralized by addition of ions. Energy minimization was performed using the steepest descent method of 10,000 steps, followed by the conjugate gradient method for 10,000 steps. NVT equilibration was done at 300 K and 100 ps of the run, followed by NPT equilibration of 100 ps. Finally, the production MD run was performed for 20 ns, whereas for TEX19 model, MD simulation was carried out at 30 ns.

### 2.4 Prediction of the pathogenic effects and disease‐related of SNPs

Different software were used for prediction of the effect of missense single nucleotide polymorphisms (SNPs) on the structure and function of the *TEX19* gene. A total of 102 missense *TEX19* SNPs obtained from dbSNP and screened by Polymorphism Phenotyping 2 (PolyPhen‐2) for possible damaging effect on the protein. Sorting Tolerant From the Intolerant (SIFT) server was used for the prediction of the deleterious effect of mutations. For the prediction of disease‐related SNPs, we used Predictor of human Deleterious Single‐Nucleotide Polymorphisms (PhD‐SNP), and SNPs&GO servers. Project HOPE webserver was used to analyze the effect of single point mutation on protein structure.

### 2.5 Prediction of amino acid conservation

Amino acid conservation among different related proteins was predicted by ConSerf server. BioEdit version 2.7.5 (Hall, 1999) was used for multiple sequence alignment and prediction of conserved sequences.

## 3 RESULTS AND DISCUSSION

### 3.1 Validation of the model

**FIGURE 1:**
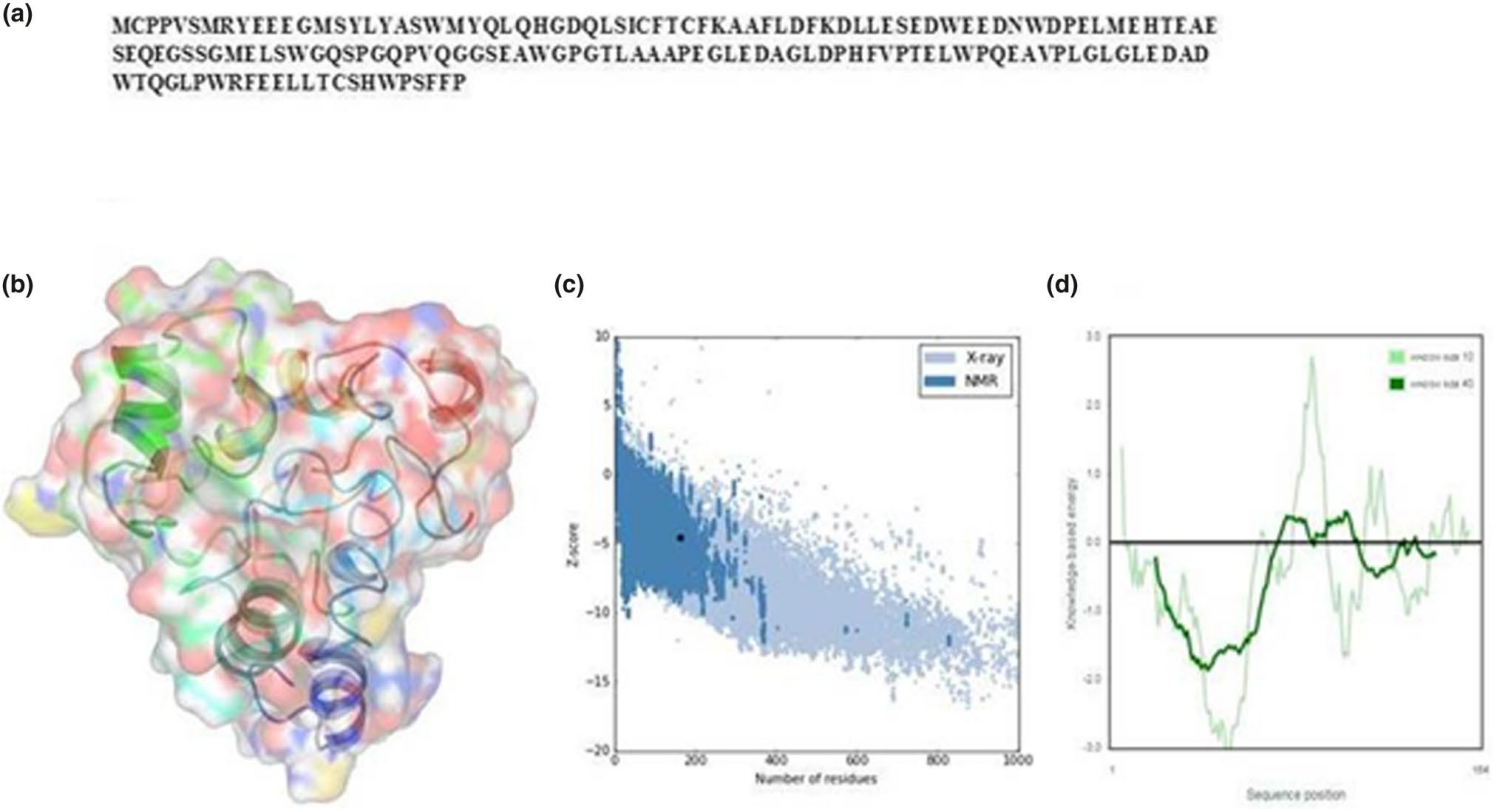
3D structure of TEX19 protein. (a) The protein sequence of TEX19. (b) 3D structure. (c) Z‐scores of all protein chains in PDB, which are determined by X‐ray crystallography (light blue) or NMR spectroscopy (dark blue). (d) Validation of the developed model

The generated model of TEX19 by I‐TASSER was done according to the template of *Streptomyces* *castaneoglobisporus* tyrosinase (1WX2) and was then validated by PROSA statistic (Figure 1). The model had an averaged Z score of −4.7, Z‐score provides an estimate of the absolute quality of a model by relating it to reference structures solved by X‐ray crystallography (Gupta et al., 2017). All these results indicated that the helicase protein model was valid.

### 3.2 Molecular docking

**FIGURE 2:**
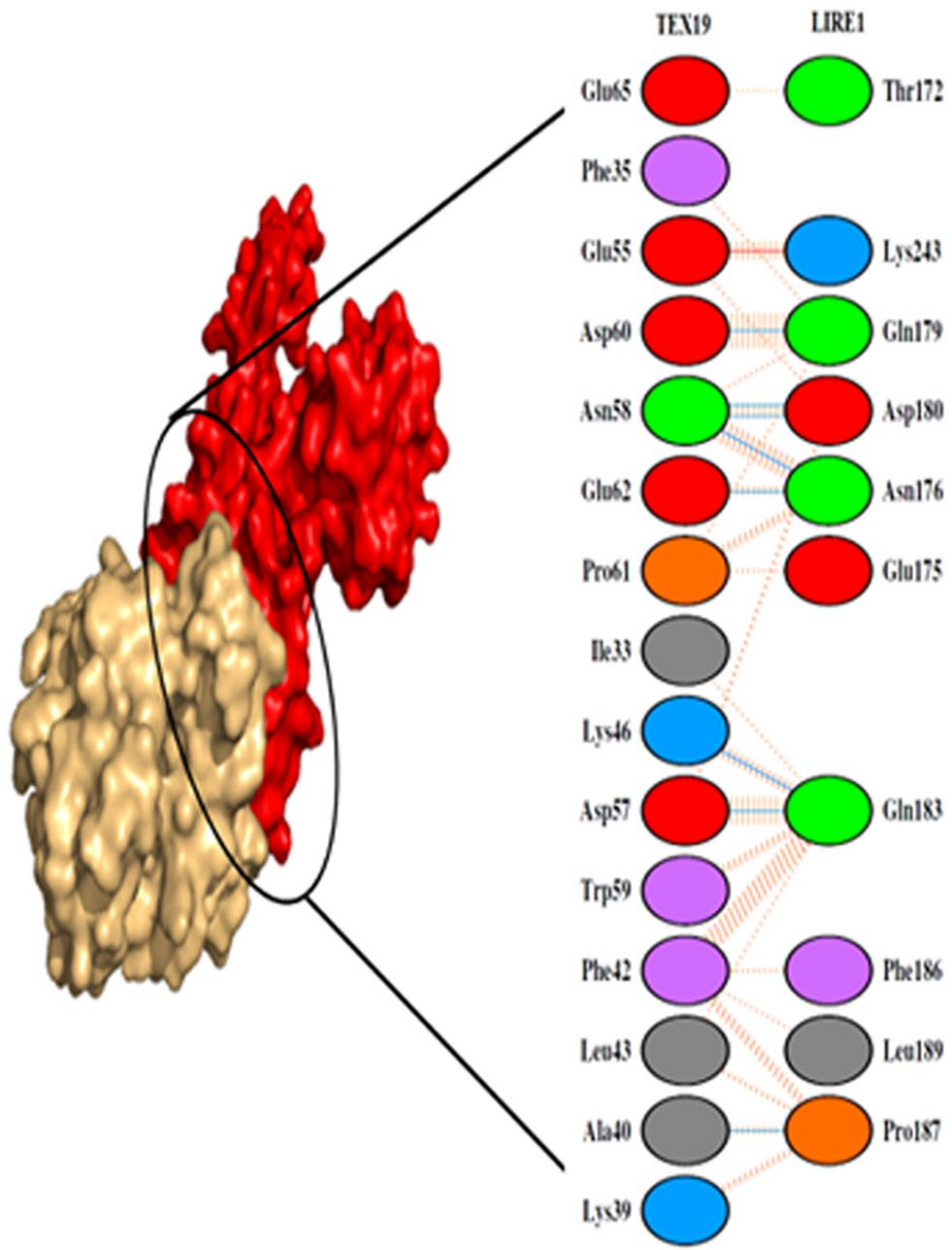
Dimplot showing the interaction between the amino acids of TEX19 and LIRE1 proteins (PDB ID: 2W7A)

Figure 2 shows the interaction pattern between TEX19 and LIRE1 proteins. Ten amino acids of TEX19 were found to stabilize the complex through the hydrogen bonding (Table 1). Hydrogen bonding is an interaction between ligand and its protein, which results in specificity and directionality to the interaction that is a fundamental aspect of molecular recognition (Itoh et al., 2019; Pace et al., 2014). Strong interaction was formed with very low binding free energy (−129.22 ± 4.5) for LIRE1 and TEX 19 proteins. Binding leads to the formation of complexes which are formed and broken depending upon several environment or external factors (Gohlke et al., 2003). The protein interactions have great significance in biology, mainly governed by the van der Waals interactions, electrostatic interactions, hydrogen bonding. A direct correlation has been reported between binding affinity and the buried surface area between a protein interface (Chen et al., 2013).

**TABLE 1:** Hydrogen bonding between the TEX19 and LIRE1 protein

S. No	TEX19	LIRE1	Distance (Å)
1	LYS 39	LEU189	2.73
2	ALA 40	PRO187	2.82
3	LYS 46	ASP 180	2.69
4	LYS 46	GLN183	2.80
5	GLU55	LYS243	2.63
6	ASP 57	GLN183	2.86
7	ALA 58	ASP180	2.97
8	ASP 60	GLN179	2.78
9	GLU 62	ASN176	2.82
10	GLU 65	ASN176	3.05

### 3.3 Molecular dynamics simulation

**FIGURE 3:**
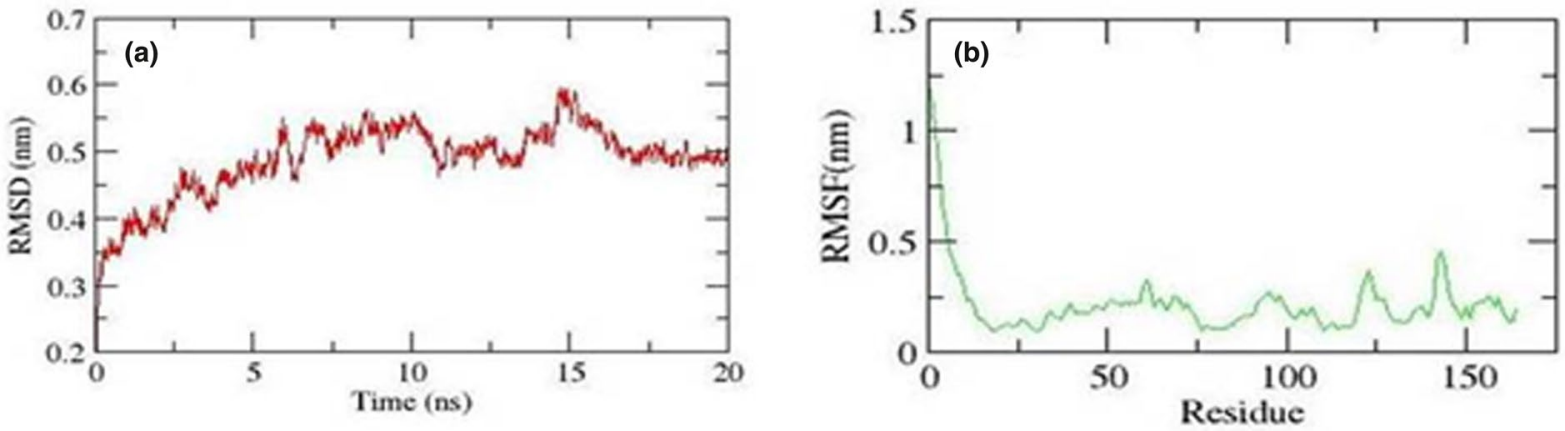
Molecular Dynamic Simulation of TEX19 and TIRE1 complex

The stability and properties of the docking formed complex was studied by explicit solvent MD simulation. The root means square deviation (RMSD) analysis not only reflects the change of protein backbone versus simulation time but also indicates the divergence of the structure. The RMSD of the complex became stable at 15 ns. The RMSD value of modeled helicase was 0.45 nm (Figure 3a). The values of RMSD also indicate the identification of appropriate interaction sites for both proteins. The root means square fluctuation (RMSF) reflects the mobility of a certain residue around its mean position, which is another tool for studying the dynamic stability of the system. Although there were some deviations among the trajectories (Especially in loop region), the present data suggested that fewer fluctuations, which further highlighted the reliability of the model structure (Figure 3b). The simulation results showed that TEX19 could bind to LIRE1 protein. This could also help in preventing the mobilization of LINE‐1 retrotransposons as was experimentally proved in mice (MacLennan et al., 2017). This could predict TEX19 potential in maintaining the trans‐generational stability of the human genome similar to the role played by TEX19.1 in mice (MacLennan et al., 2017).

### 3.4 Prediction of SNPs affect the TEX19 structure and function

Table 2 shows the predicted effect of missense SNPs on TEX19 protein structure and function. From 102‐screened missense SNPs, 37 were probably damaging, 18 were possible damaging, and 47 were benign. Out of 102 SNPs only six (S15C, C34Y, W147R, R8 W, C37S, and W141R) were predicted by PhD server to be disease‐related, while only two (C34Y, C37S) predicted by SNPs and GO to be disease‐related. From 37 probably damaging SNPs, 29 predicted deleterious to TEX19 protein. Three probably damaging polymorphisms (F35S, P61R, and E55L) with Minor Allele Frequency (MAF) 0.000004/1, located at the binding site of LIRE1 were observed, the presence of these variants at the binding site of this TEX19 could affect its activity and binding affinity. The substitution of Phenylalanine (F) into a Serine (S) at position 35, could disturb this binding site, because the mutant residue is smaller than the wild‐type residue, and the wild‐type residue is more hydrophobic than the mutant residue, in addition to Phe35 located at highly conserved region so the differences in amino acid properties can disturb this region and thus disturb its function. The substitution of proline (P) into arginine (R) at position 61 could disrupt the structure and binding cavity of protein, due to the fact that the mutant residue is bigger than the wild‐type residue and the wild‐type residue charge was neutral. In contrast, the mutant residue charge is positive, and the wild‐type residue is more hydrophobic than the mutant. Besides, prolines are known to be very rigid and therefore induce a special backbone conformation which might be required at this position, thereby the loss of proline at this point could disturbing the local structure. The mutation of a Glutamic Acid (E) into a leucine (L) at position 55 could disrupt the pocket used for binding of the LIRE1. Due to difference in charge, size, and hydrophobicity, these variations can result in loss of hydrogen bonds and/or disturb correct protein folding.

**TABLE 2:** Predicted effect of missense SNPs on TEX19 protein structure and function

SNP ID	AA	PolyPhen−2 (score)	PhD	SNPs and GO	SIFT
rs377629628	M1T	Probably damaging (0.998)	Neutral	Neutral	Deleterious
rs371327683	P4L	Probably damaging (1.000)	Neutral	Neutral	Deleterious
rs761018318	P4A	Probably damaging (1.000)	Neutral	Neutral	Deleterious
rs900962762	S6G	Probably damaging (0.969)	Neutral	Neutral	Deleterious
rs1245990387	S6R	Probably damaging (0.997)	Neutral	Neutral	Deleterious
rs868177850	R8W	Probably damaging (0.999)	Disease	Neutral	Deleterious
rs1318138374	H27Q	Probably damaging (0.998)	Neutral	Neutral	Deleterious
rs150740969	C34Y	Probably damaging (0.993)	Disease	Disease	Deleterious
rs367711836	F35S	Probably damaging (0.999)	Neutral	Neutral	Deleterious
rs1257967420	C37S	Probably damaging (0.999)	Disease	Disease	Deleterious
rs1185108733	A41V	Probably damaging (1)	Neutral	Neutral	Deleterious
rs1259349893	A41S	Probably damaging (1)	Neutral	Neutral	Deleterious
rs116114329	E50A	Probably damaging (1.000)	Neutral	Neutral	Deleterious
rs1156477833	E50D	Probably damaging (1)	Neutral	Neutral	Deleterious
rs1599669625	E55L	Probably damaging (0.997)	Neutral	Neutral	Deleterious
rs1385130699	D60N	Probably damaging (0.998)	Neutral	Neutral	Deleterious
rs1331784175	P61R	Probably damaging (0.962)	Neutral	Neutral	Tolerated
rs1266481991	L63M	Probably damaging (0.987)	Neutral	Neutral	Tolerated
rs761225499	W83R	Probably damaging (0.991)	Neutral	Neutral	Tolerated
rs1390892071	W83C	Probably damaging (0.998)	Neutral	Neutral	Tolerated
rs1319739886	P90H	Probably damaging (0.99)	Neutral	Neutral	Tolerated
rs777468964	Q92H	Probably damaging (0.965)	Neutral	Neutral	Tolerated
rs147220016	G93W	Probably damaging (0.997)	Neutral	Neutral	Deleterious
rs140570015	G99R	Probably damaging (0.979)	Neutral	Neutral	Deleterious
rs1372613314	A104E	Probably damaging (0.997)	Neutral	Neutral	Tolerated
rs1184019063	P121S	Probably damaging (1)	Neutral	Neutral	Deleterious
rs1403014449	P126S	Probably damaging (1)	Neutral	Neutral	Deleterious
rs1599669625	Q127L	Probably damaging (0.979)	Neutral	Neutral	Deleterious
rs1479032497	P131L	Probably damaging (0.99)	Neutral	Neutral	Deleterious
rs200970555	G135S	Probably damaging (0.998)	Neutral	Neutral	Deleterious
rs1439503805	W141R	Probably damaging (1)	Disease	Neutral	Deleterious
rs1276048234	Q143H	Probably damaging (1)	Neutral	Neutral	Deleterious
rs771614199	G144C	Probably damaging (0.997)	Neutral	Neutral	Deleterious
rs759919288	W147R	Probably damaging (1)	Disease	Neutral	Deleterious
rs933781675	W158C	Probably damaging (1)	Disease	Neutral	Deleterious
rs767149161	P159S	Probably damaging (0.979)	Neutral	Neutral	Tolerated

### 3.5 Conservation score

**FIGURE 4:**
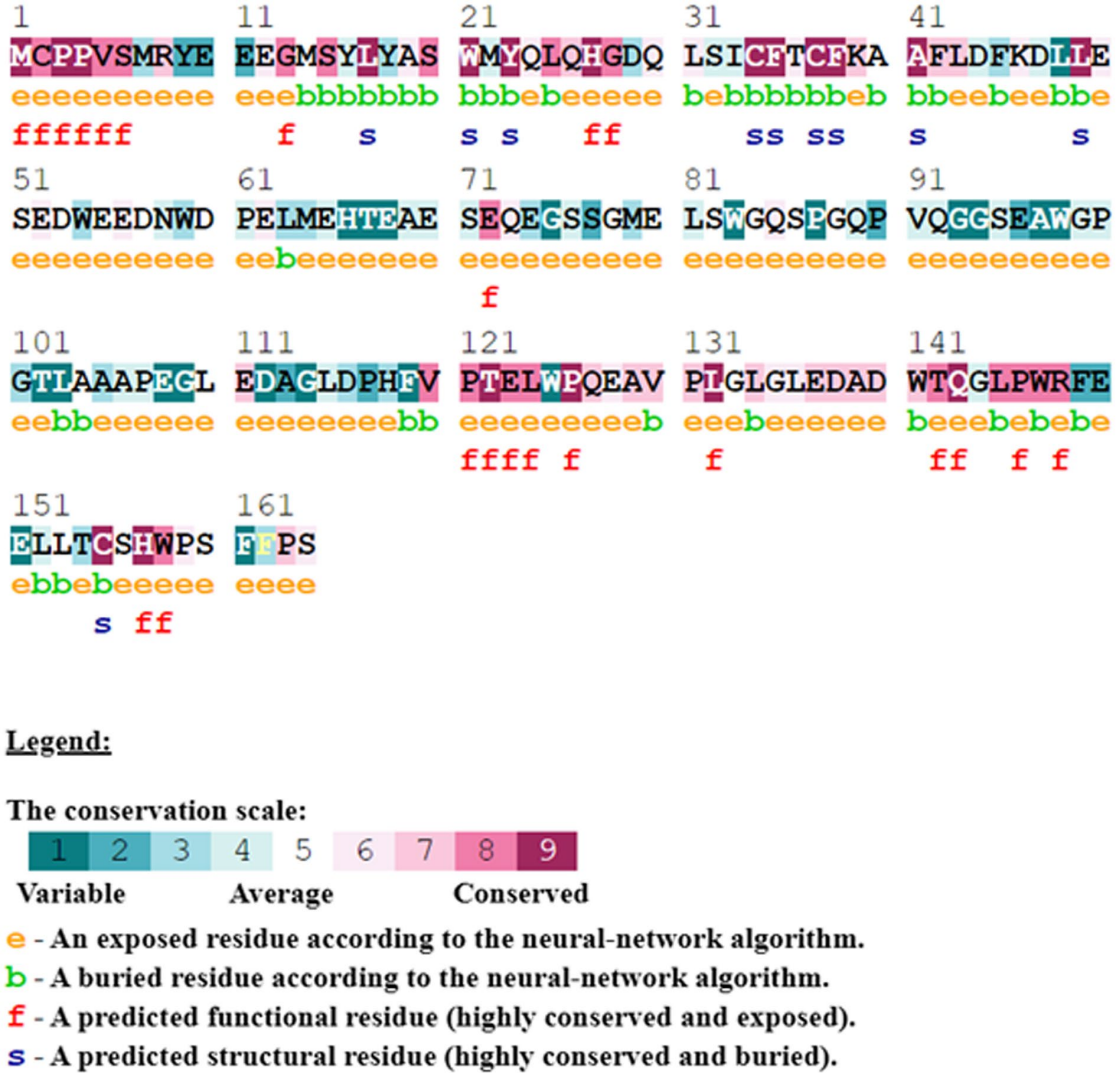
TEX19 amino acid sequences conservation score among different species

**FIGURE 5:**
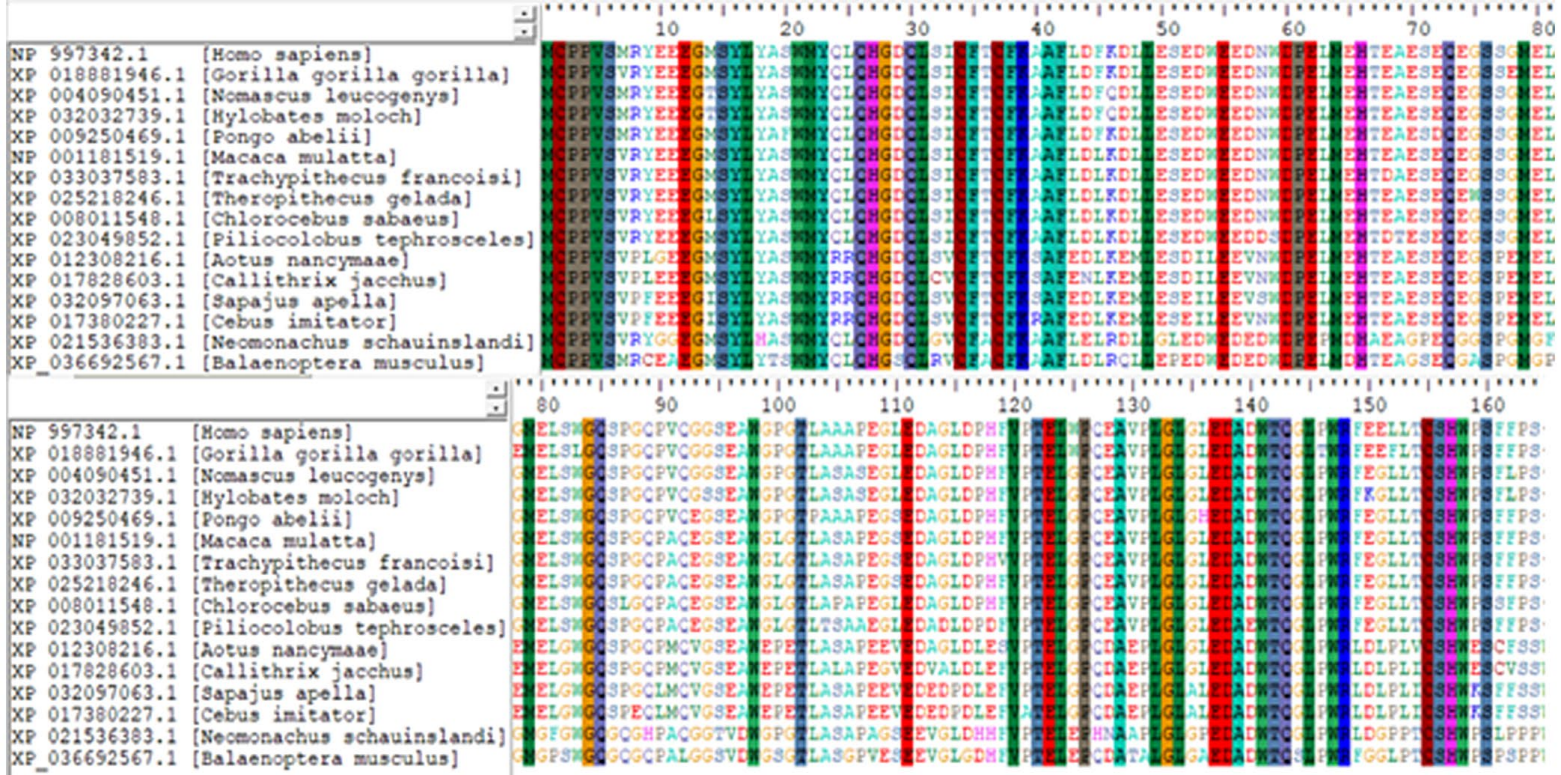
Multiple sequence alignment of TEX19 gene sequences of different species showing conserved sequences in vertical lines

Residues of TEX19 (LYS 39, GLU 62) showing hydrogen bonds with LIRE1 were among conserved sequences. Phe35 of TEX19 which have an interaction with LIRE1 predicted among highly conserved buried residues (Figures 4 and 5).

## 4 CONCLUSIONS

In silico methods such as docking and molecular dynamic (MD) simulations are used to find the correct conformation of a ligand and its receptor and have been used previously in drug design (Bissaro et al., 2020; Garofalo et al., 2020; Maximov et al., 2020; Salmaso & Moro, 2018). In this study we performed docking and MD simulations methods was to evaluate the interaction between TEX19 and LIRE1 proteins and identified an alternative binding pocket in the TEX19 protein based on the consensus binding site. For this protein, 10 amino acids of TEX19 were found to stabilize the complex through the hydrogen bonding. A total of 37 missense variants were predicted to play a probably damaging role for the protein, three of them (F35S, P61R, and E55L) located at the binding site of LIRE1 and could disturb this binding affinity. The F35S located at highly conserved region, mutations at highly conserved region could severely affect protein function and structure (Liu et al., 2014; Stefancsik et al., 1998).

## CONFLICTS OF INTEREST

The authors have declared no conflict of interest.

## AUTHOR CONTRIBUTIONS

Conceptualization, O.R.A, F.A., and Y.H.; methodology, F.A., Y.H., H.A., and N.A.; software, Y.H., H.A., and N.A.; validation, F.A., Y.H., H.A., and N.A.; formal analysis, Y.H., H.E.A., and N.A.; investigation, Y.H., H.A., L.M., S.A., and N.A.; resources, F.A., Y.H., H.A., and N.A.; data curation, F.A., Y.H., H.A., L.M., and N.A.; writing—original draft preparation, O.M.A., F.A., and Y.H.; writing—review and editing, F.A., Y.H., H.A., S.A., L.M., N.A.,O.R.A., H.E.A., and O.M.A.; funding acquisition, F.A., N.A., S.A., and O.R.A.

## Data Availability

The data that support the findings of this study are available on request from the corresponding author.
